# Adult congenital adrenal hyperplasia presenting with severe clitoromegaly and distal vaginal stenosis: A reconstructive surgical case report

**DOI:** 10.1016/j.eucr.2026.103411

**Published:** 2026-03-17

**Authors:** Marwa Alatawi, Magdoleen Barry, Raed Almannie, Abdulaziz Althunayan, Abdulrahman Ahmed Albassam, Lateefa Othman Aldakhil

**Affiliations:** a Obstetrics and Gynecology Department, College of Medicine, King Saud University, Riyadh, 11362, Saudi Arabia; b Urology Department, College of Medicine, King Saud University, Riyadh, 11362, Saudi Arabia; c Department of Pediatric and Surgery, College of Medicine, King Saud University, Riyadh, 11362, Saudi Arabia

**Keywords:** Congenital adrenal hyperplasia, Clitoromegaly, Urogenital sinus, Vaginoplasty, Neurovascular-sparing clitoroplasty, Reconstructive surgery, Adult CAH

## Abstract

Congenital adrenal hyperplasia (CAH) usually causes genital virilization managed in early childhood; adult presentation with severe clitoromegaly and persistent urogenital sinus is rare. We report a 30-year-old woman with CAH presenting with clitoromegaly and menstrual flow during urination only.MRI demonstrated a short urogenital sinus with distal vaginal stenosis and marked clitoral enlargement. Multidisciplinary reconstruction included neurovascular-sparing clitoroplasty, complete urethrovaginal separation, distal vaginoplasty, and labial reconstruction. Postoperatively, the patient achieved normal urinary and menstrual function, preserved clitoral sensation, and satisfactory cosmetic results without complications. This case demonstrates the feasibility of complex nerve-sparing urogenital reconstruction in adult CAH patients.

## 1 Introduction

Congenital adrenal hyperplasia (CAH) is the most common cause of virilization in individuals with a 46,XX karyotype, most often due to 21-hydroxylase deficiency.^1^ Excess prenatal androgen exposure results in a spectrum of genital anomalies, including clitoromegaly, labioscrotal fusion, and formation of a urogenital sinus, where the urethra and vagina share a common channel.^2^

In most cases, CAH-related genital anomalies are diagnosed and surgically managed in infancy or early childhood. Feminizing genitoplasty aims to achieve acceptable genital appearance while preserving neurovascular integrity and allowing normal urinary, menstrual, and future sexual function.^3^^,^^4^

Although surgical techniques have evolved toward neurovascular-sparing approaches, long-term outcomes remain variable, with vaginal stenosis, urinary dysfunction, and sensory impairment reported in a subset of patients 5, 6, 7, 8. Comparative long-term outcome data remain limited, and consensus regarding optimal surgical techniques continues to evolve.^9^^,^^10^

Adult presentation of untreated or residual CAH-related genital anomalies is exceptionally rare, as most patients undergo early definitive management.^11^ When adults do present, manifestations may include persistent clitoromegaly, vaginal stenosis, or abnormal urinary or menstrual flow. Only isolated cases of adult genital reconstruction have been reported, and guidance on management remains limited 12, 13, 14, 15.

We report a rare adult case of CAH presenting with severe clitoromegaly, distal vaginal stenosis, and menstrual outflow through the urinary tract due to a persistent urogenital sinus. This case highlights the diagnostic challenges of delayed presentation and demonstrates the feasibility of multidisciplinary adult genital reconstruction using neurovascular-sparing clitoroplasty and urogenital sinus.

## 2 Case presentation

**Fig. 1:**
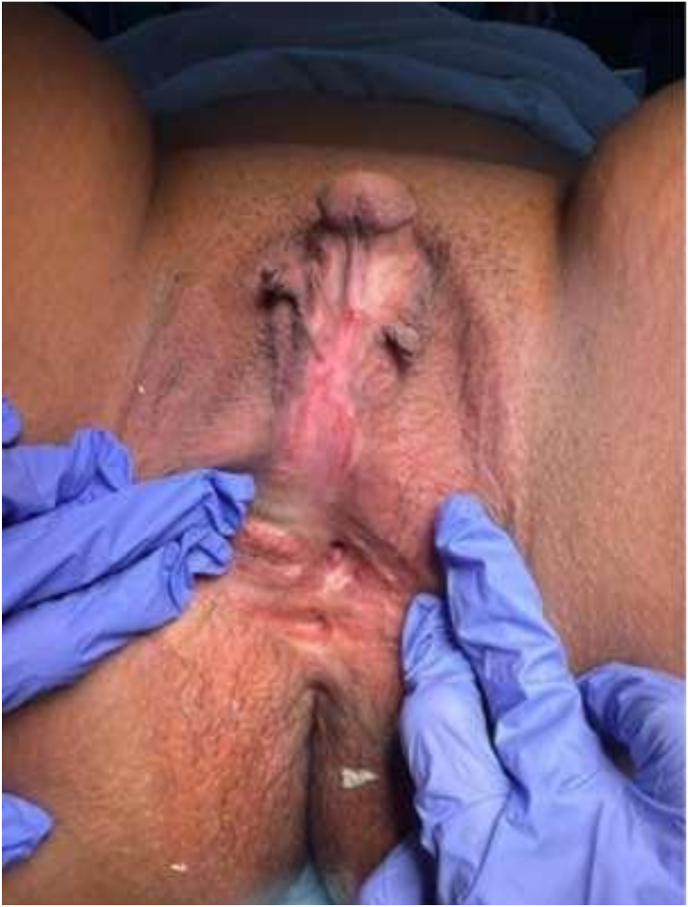
Clitoral shaft measuring approximately 6.6 cm in length and 3 cm in width.

**Fig. 2:**
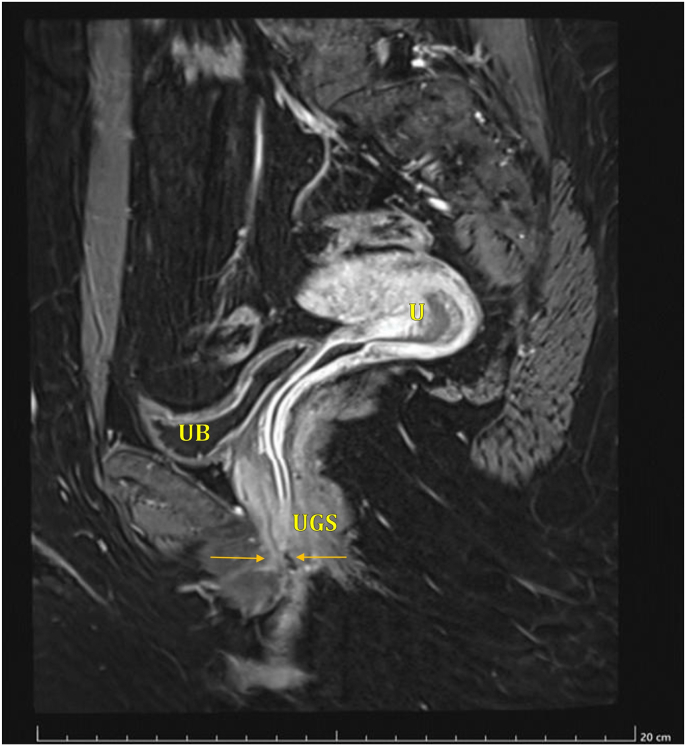
Oblique para sagittal fat suppressed enhanced T1WI showing short urogenital sinus tract of 1 cm with narrowed distal 3 cm vagina (from introitus). The site of confluence point between urethra and narrowed vagina (opposing arrows) (see schematic diagram figure) UB=Urinary bladder, U=Uterus, UGS=Urogenital sinus.

A 30-year-old Saudi female, known case of congenital adrenal hyperplasia (CAH) since infancy, presented with a history of menstrual bleeding with urination. She reported that menses appeared simultaneously with urine, suggesting an abnormal urogenital communication or distal obstruction. She had undergone genital corrective surgery for Ambiguous genitalia during infancy the exact details of the procedure were unavailable. She later underwent foramen magnum decompression for Chiari malformation during adolescence. Examination under anesthesia revealed severe clitoromegaly measuring approximately 6.6 cm × 3 cm, small labia minora, and partially fused labia majora. A common urogenital sinus was located 6 cm below the glans. No palpable gonads or inguinal masses were found (Fig. 1). Hormonal evaluation revealed elevated ACTH and 17-hydroxyprogesterone levels with low-normal aldosterone, consistent with poorly controlled CAH. Pelvic magnetic resonance imaging (MRI) was performed to delineate the anatomy of the clitoris, vagina, urethra, and urogenital sinus prior to surgical intervention. Oblique parasagittal fat-suppressed contrast-enhanced T1-weighted images demonstrated a short urogenital sinus tract measuring approximately 1 cm, with a narrowed distal vaginal segment extending approximately 3 cm from the introitus. The exact confluence point between the urethra and the narrowed distal vagina was clearly visualized. The urinary bladder and uterus were normal in morphology (Fig. 2).

Mid-sagittal T2-weighted images revealed marked clitoromegaly. Axial T2-weighted images showed significant enlargement of both corpora cavernosa with visualization of the vestibular bulbs. An artificial opening near the left base of the clitoris likely related to prior childhood surgery was identified and represented the common outlet for both urine and menstrual flow. These MRI findings confirmed the presence of a residual urogenital sinus associated with distal vaginal stenosis and severe clitoromegaly, and were critical in guiding multidisciplinary surgical planning.

After obtaining written informed consent, the patient underwent multidisciplinary genital reconstructive surgery involving urogynecology, urology, and pediatric surgery teams.

Preoperative optimization was completed in coordination with the endocrinology service. Surgical goals were to achieve functional separation of the urinary and genital tracts, reduce clitoral size while preserving neurovascular integrity, and restore anatomically and aesthetically acceptable external genitalia.

Under general anesthesia, the patient was positioned in lithotomy. A Foley catheter was inserted to delineate the urethra. Intraoperative cystoscopy was performed to assess the urethra, bladder neck, and site of urethrovaginal confluence, confirming the anatomical findings demonstrated on preoperative MRI. The perineum was prepared and draped in a sterile fashion. Intraoperative markings were used to outline the clitoral shaft, prepuce, and labial folds.

Examination revealed marked clitoromegaly, with a clitoral shaft measuring approximately 6.6 cm in length and 3 cm in width, covered by redundant preputial skin and associated with hypoplastic labia minora. A common urogenital sinus opening was identified approximately 6 cm distal to the glans, consistent with fusion of the urethral and vaginal orifices. The clitoral glans was broad and well vascularized (Fig. 1).

**Fig. 3:**
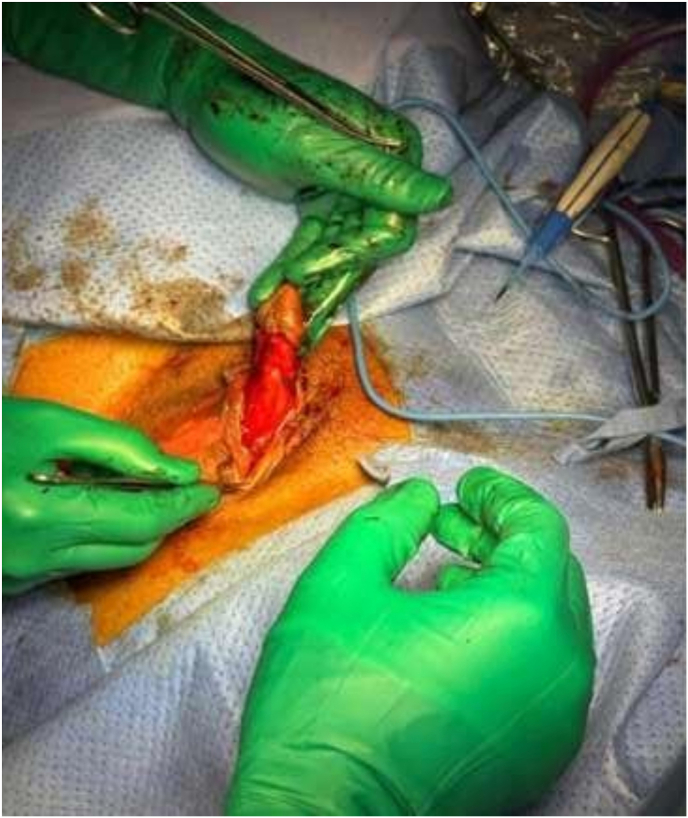
Degloving of the clitoral shaft with ventral and lateral dissection, preserving the dorsal midline and protecting the dorsal neurovascular bundle.

A subcoronal circumferential incision was made, followed by careful degloving of the clitoral shaft. Dissection was performed between the skin–subcutaneous tissue and the tunica albuginea of the corpora cavernosa. The dorsal neurovascular bundle was identified and preserved along its entire length. The glans was maintained on a ventral vascular pedicle to ensure adequate perfusion and sensory preservation see Fig. 3.

The paired corpora cavernosa were dissected in the midline and divided proximally near the pubic arch. Excess distal corporal tissue was excised in a controlled manner, guided by a Hegar dilator to achieve appropriate clitoral length and projection. The proximal corporal stumps were secured with figure-of-eight 3-0 PDS sutures for hemostasis. The ventral tunica albuginea was preserved to provide structural support (Fig. 3).

The preserved glans was repositioned over the reduced corporal stumps and fixed using interrupted 5-0 PDS sutures, creating a natural-appearing neo-clitoris. Adequate vascularity was confirmed intraoperatively by capillary refill and tactile response.

**Fig. 4:**
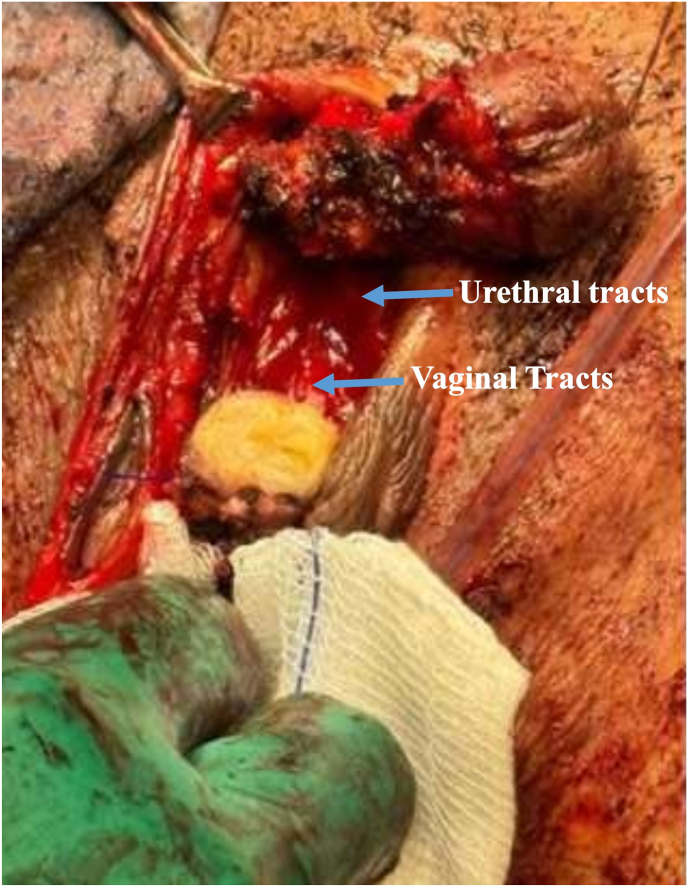
Mobilization and dissection of the urogenital sinus with separation of urethral and vaginal tracts.

Attention was then directed to the urogenital sinus. The common channel, measuring approximately 1 cm, was circumferentially mobilized to the level of bifurcation. The urethra and vagina were carefully separated with meticulous dissection to avoid injury to the bladder neck and distal vaginal wall Fig. 4.

Reconstruction was performed in layers. The urethral wall was repaired using interrupted 4-0 Vicryl sutures. The vaginal wall was reconstructed in two layers using 3-0 Vicryl sutures. A fatty interpositional graft was placed between the urethral and vaginal suture lines to reduce the risk of urethrovaginal fistula formation (Fig. 4).

Distal vaginoplasty was performed using local mucosal advancement flaps to optimize vaginal caliber. The dorsal preputial skin was divided longitudinally and fashioned into bilateral triangular flaps, which were rotated medially and inferiorly to reconstruct the labia minora. Fine interrupted 5-0 Monocryl sutures were used to achieve symmetry and satisfactory cosmetic outcome.

Hemostasis was confirmed. A 14-Fr Foley catheter was inserted for urinary drainage, and a light vaginal pack was placed to support the reconstructed vaginal walls. A sterile dressing was applied. Total operative time was approximately 5 h, with minimal blood loss and no intraoperative complications.

At the completion of the procedure, the external genitalia demonstrated a well-proportioned clitoral glans with natural projection, clearly separated urethral and vaginal openings, and symmetrical labia minora.

The patient received broad-spectrum intravenous antibiotics (meropenem for three days) due to a preoperative multidrug-resistant *Escherichia coli* urinary tract infection. Standard postoperative analgesia and venous thromboembolism prophylaxis were administered.

**Fig. 5:**
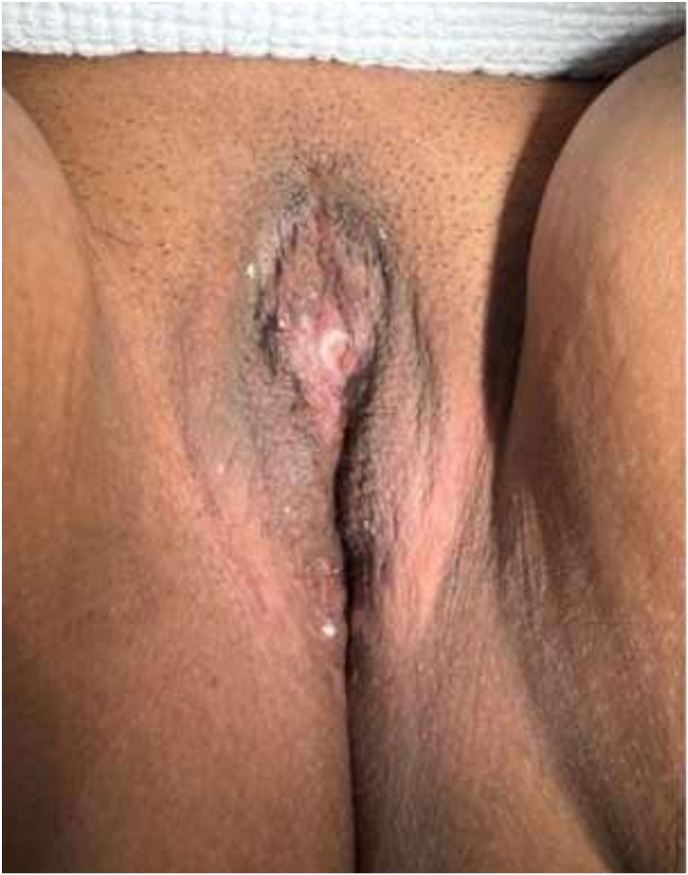
Three-month postoperative result demonstrating excellent healing, patent vaginal canal, and preserved clitoral contour.

The Foley catheter was maintained for two weeks and removed after confirmation of satisfactory healing and unobstructed urinary flow Fig. 5.

At three-month follow-up, the patient demonstrated a patent vaginal canal, normal urinary stream, preserved clitoral vascularity and sensation, and no evidence of infection, fistula, or stenosis.

## 3 Patient perspective

For many years, I felt different and embarrassed about my body, and I was too shy to seek medical help. When my period started coming out with urine, I felt scared and very alone. After the surgery, I finally feel normal. I can pass urine and menstruate separately, and I feel like a woman now. This has given me confidence, relief, and peace of mind, and I am truly grateful to the team who changed my life.

## 4 Discussion

Adult presentation of congenital adrenal hyperplasia (CAH) with severe clitoromegaly and a persistent urogenital sinus is rare, as most patients undergo corrective surgery during infancy or early childhood.^3^^,^^4^ When delayed presentation occurs, reconstruction is more challenging due to prolonged androgen exposure, altered anatomy, fibrosis from prior surgery, and reduced tissue elasticity, limiting the applicability of pediatric outcome data to adult patients.^5^^,^^6^

In the present case, menstrual flow occurring during urination was caused by a persistent urogenital sinus associated with distal vaginal stenosis. Such symptoms may be misinterpreted as primary lower urinary tract pathology, leading to delayed diagnosis. This highlights the importance of careful anatomical evaluation in adult CAH patients presenting with atypical urinary or menstrual complaints.

Preoperative pelvic magnetic resonance imaging (MRI) played a key role in delineating the urogenital anatomy, identifying the length of the common channel, and defining the urethrovaginal confluence. MRI is particularly valuable in adults with previous genital surgery, as it allows visualization of fibrosis and distorted tissue planes and improves operative safety.^8^ Intraoperative cystoscopy was used as an adjunct to confirm urethral and bladder neck anatomy prior to definitive urethrovaginal separation.

Neurovascular-sparing clitoroplasty is now considered the standard surgical approach in CAH reconstruction. Preservation of the dorsal neurovascular bundle and ventral vascular pedicle allows effective reduction of erectile tissue while maintaining clitoral sensation and vascularity 5, 6, 7^,^^9^. The favorable early functional and cosmetic outcomes in this patient support the use of nerve-sparing techniques even in delayed adult presentations.

Reconstruction of the urogenital sinus remains technically demanding, particularly in adult patients. Layered urethrovaginal repair with interposed tissue was used to reduce the risk of urethrovaginal fistula formation and achieve durable anatomical separation.^8^^,^^10^ Adjunctive distal vaginoplasty and labial reconstruction further optimized vaginal caliber and external genital appearance.

Successful management of adult CAH reconstruction requires a multidisciplinary approach involving urology, gynecology, pediatric surgery, and endocrinology. With detailed anatomical assessment, hormonal optimization, and meticulous surgical technique, complex urogenital anomalies can be safely corrected in adulthood with satisfactory functional and cosmetic outcomes 11, 12, 13, 14, 15.

## 5 Conclusion

Adult congenital adrenal hyperplasia may present with complex genital anomalies that require individualized and technically demanding reconstruction. Neurovascular-sparing clitoroplasty combined with layered urogenital sinus reconstruction can successfully restore anatomy, function, and cosmetic outcomes. Optimal results depend on meticulous surgical planning, appropriate hormonal optimization, and a coordinated multidisciplinary approach to ensure long-term functional and psychological well-being.

## CRediT authorship contribution statement

**Marwa Alatawi:** Writing – review & editing, Writing – original draft, Methodology, Data curation, Conceptualization. **Magdoleen Barry:** Writing – review & editing, Writing – original draft, Methodology, Data curation, Conceptualization. **Raed Almannie:** Writing – review & editing, Investigation, Data curation. **Abdulaziz Althunayan:** Writing – review & editing. **Abdulrahman Ahmed Albassam:** Writing – review & editing, Investigation, Data curation. **Lateefa Othman Aldakhil:** Writing – review & editing, Validation, Supervision, Investigation, Data curation, Conceptualization.

## Patient consent

Written informed consent was obtained from the patient for publication of this case report and accompanying images.

## Funding

No external funding was received for this study.

## Declaration of competing interest

The authors declare no conflict of interest.
